# Chiral Nanotubes

**DOI:** 10.3390/nano7070167

**Published:** 2017-07-04

**Authors:** Andrea Nitti, Aurora Pacini, Dario Pasini

**Affiliations:** 1 Department of Chemistry, University of Pavia, Viale Taramelli, 12-27100 Pavia, Italy; andrea.nitti01@universitadipavia.it (A.N.); au.pacini@gmail.com (A.P.); 2 INSTM Research Unit, University of Pavia, Viale Taramelli, 12-27100 Pavia, Italy

**Keywords:** chirality, nanotubes, three-dimensional (3D) assembly, supramolecular polymers, anisotropic materials

## Abstract

Organic nanotubes, as assembled nanospaces, in which to carry out host–guest chemistry, reversible binding of smaller species for transport, sensing, storage or chemical transformation purposes, are currently attracting substantial interest, both as biological ion channel mimics, or for addressing tailored material properties. Nature’s materials and machinery are universally asymmetric, and, for chemical entities, controlled asymmetry comes from chirality. Together with carbon nanotubes, conformationally stable molecular building blocks and macrocycles have been used for the realization of organic nanotubes, by means of their assembly in the third dimension. In both cases, chiral properties have started to be fully exploited to date. In this paper, we review recent exciting developments in the synthesis and assembly of chiral nanotubes, and of their functional properties. This review will include examples of either molecule-based or macrocycle-based systems, and will try and rationalize the supramolecular interactions at play for the three-dimensional (3D) assembly of the nanoscale architectures.

## 1. Introduction

**Figure 1:**
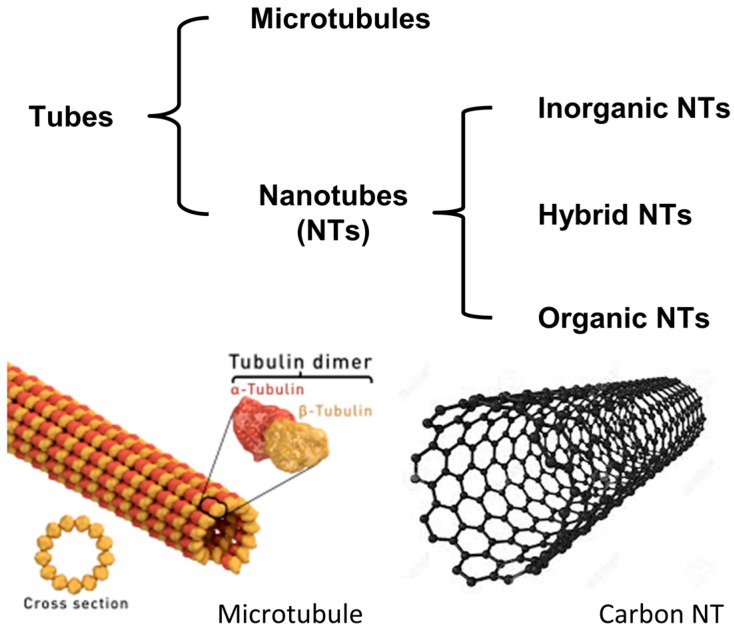
Classification of tubular objects, and a schematic representation of a biological tubule (micrometer scale), and of a single-walled carbon NT (SWCNT) (nanometer scale).

The organization of small molecules, macrocycles and polymers into tubes with hollow cavities is a key structural feature of living systems, as exemplified by cytoplasmic microtubules [1]. The term tubule refers to a small tube or canal and it is ubiquitous in biological and medical dictionaries. In general, tubes can be grouped into two principal categories, depending on tube diameter: (a) nanoscale tubes (nanotubes); and (b) microscale tubes (microtubes or tubules). Nanotubes (NTs), which present a diameter in the range several to 100 nanometers and a length-to-diameter ratio that can be greater than 10^6^, have potential for technological applications starting from medicine and biology, and passing through electronics, optics and sensing. Depending on their nature, they can be classified as inorganic NTs [2], organic NTs and hybrid NTs (Figure 1). Hybrid NTs are essentially formed by complementary organic and inorganic structures, and they have been recently reviewed [3]. The prototypical organic NT are carbon NT, and in particular single-walled carbon NTs (SWCNTs), which are cylindrical molecular structures, often considered as rolled-up graphene sheets. Depending on the angles with which the graphene sheet rolls up, SWCNTs can have different chirality and handedness. Their typical industrially adopted synthesis include arc discharge, laser ablation, and chemical vapor deposition techniques [4,5,6], but they invariably lead to a mixture of nanotubes with a broad distribution in diameter and chirality. The unique electronic and optical properties of SWCNTs, such as high surface area, high current capacity, and mechanical and chemical properties, can be significantly influenced by their dimensions and three-dimensional (3D) structure. Obtaining homogeneous SWCNTs, i.e., the same diameter and the same enantiomeric form, is a major challenge for their large-scale applications, and has gained considerable research attention [7,8,9].

Chirality plays an important role in the materials field, influencing optical, magnetic and electrical properties such as magnetochiral circular dichroism, magnetic skyrmions in chiral magnetics and nonreciprocal carrier transport in chiral conductors [10,11,12]. Controlling the chirality of tubular nanosystems will be essential to increase their potential for targeted applications.

The self-organization of organic molecules in 3D structures is a parallel, and increasingly popular way to achieve organic NTs. Inspired by biological systems, numerous efforts have been devoted to the design of synthetic building blocks that can form such hollow nanostructures through orchestrated interplay of various noncovalent interactions, using well-established principles of crystal engineering and supramolecular chemistry. Their development has already opened a broad spectrum of opportunities and potential for applications in sensing, optics, organic electronics, and catalysis, as well as in biology and medicine [13,14,15,16].

Since the field described in the introduction is indeed too ample and varied to be described into a single short review, we will exclude research related to chiral SWCNTs. Their selective synthesis has been recently reviewed [17], and supramolecular approaches to their enantiomeric resolution have appeared [18]. Oligomers and polymers can self-organize in appropriate solvents to give folded structures (foldamers), with an interior cavity. In this case, the length of the nanotube is dictated by the dimension of the unfolded oligomer-polymer precursor. This elegant approach is too ample to be discussed here, and several thorough and authoritative reviews have already appeared [19,20,21,22,23,24,25,26]. This review will focus on progress in research on chiral NTs architectures based on small molecules (cyclic or acyclic in their initial nature), their functional properties and applications.

## 2. Discussion

This review is centered on chiral NTs, and will deal with organic NTs obtained by the assembly of chiral molecules. Chiral synthetic nanotubes have been targeted essentially by two complementary strategies: (a) stacking of covalent cyclic molecules; or (b) 3D self-assembly of smaller building blocks. Preformed covalent chiral macrocycles can offer advantages in terms of the tuning of the cavity magnitude; furthermore, the direct introduction of chirality elements into the cyclic skeleton of the macrocycles can lead to high control of the resulting helicity and directionality during the nanotube assembly. On the other end NTs formed from self-assembly of small building blocks can offer advantages in their easy and scalable synthesis. The aggregation of organic building blocks and macrocycles into the third dimension of space, to form organic nanotubes, occurs through the implementation of various noncovalent interactions, such as hydrogen bonds, *π*–*π* stacking interactions, metal mediated interactions, and multiple interactions. Although the interplay between these noncovalent forces is often in place, we have classified them separately in different subchapters. In general, the most used techniques to characterize chiral nanotubes are: (i) Scanning Electron (SEM) and Atomic Force (AFM) microscopies; and (ii) Circular Dichroism (CD) spectroscopy. The first ones are imaging techniques, useful to verify the formation and the relative morphology of the nanotubular structures: both are able to achieve resolution in the order of nanometers and mainly, the samples are prepared by solution casting onto appropriate surfaces. CD spectroscopy usually allows the gathering of fundamental information on the supramolecular structures formed and to check the chirality of the tubular superstructures, by examining the absorption bands of optically active chiral molecules and ensembles; most measurements are reported in degrees of ellipticity.

### 2.1. Hydrogen Bonds

In recent years, the construction of hydrogen-bonded tubular assemblies of peptide macrocycles has become an important area of research for the realization of biomimetic materials with useful applications both as models for biological channels and as materials with novel electronic and photonic properties [13,16,27,28,29,30]. In general, cyclic-(*D*,*L*)-α-peptides, β- and γ-peptides and peptide hybrids constitute a class of flat ring-shaped molecules forming β-sheet-like assemblies, through the intermolecular association of amide hydrogen bonding units present in the cyclic backbone. The internal diameter of the tubular structures can be varied by changing the size of cyclopeptides, making these molecules very interesting. The first example of organic NT originated by a cyclic-(*D*,*L*)-α-peptide, able to form hollow tubular structures through antiparallel β-sheet hydrogen bonding, was reported in 1993 by Ghadiri et al. [31]. The *cyclo*[-(*L*-Gln-*D*-Ala-*L*-Glu-*D*-Ala)_2_-] presents an alternating *D* and *L* arrangement of amino acid residues within the cyclopeptide backbone, forming a cyclic structure with an overall *C_2_* symmetry in enantiomerically pure form, given that enantiopure amino acid building blocks are used in the solid phase synthesis of the starting linear peptide, which is subsequently cyclized. The described net molecular chirality translates therefore to form homochiral nanotubes, which were not however further characterized from the point of view of their chiroptical properties.

**Figure 2:**
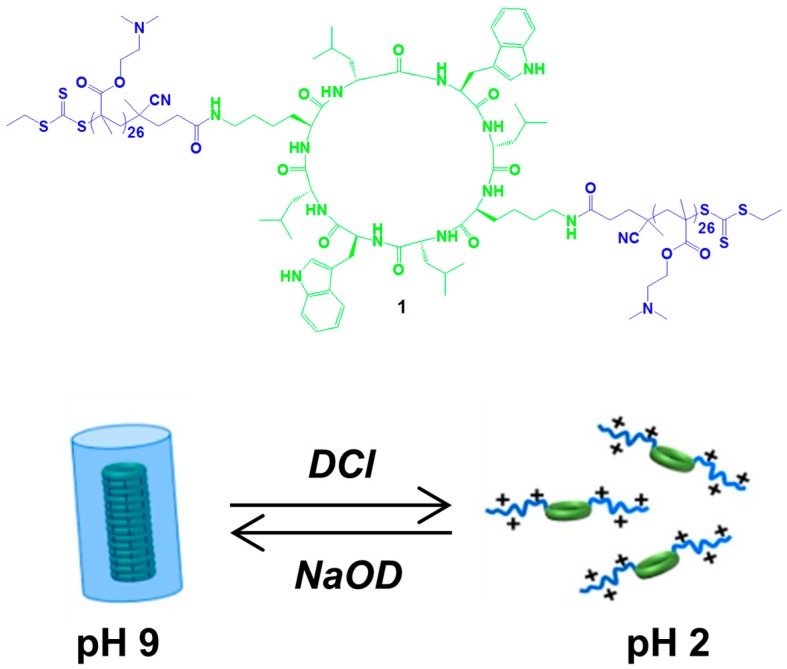
Molecular structure of the cyclopeptide-polymer conjugate **1** and illustration of pH responsiveness in the formation of NTs. Partially reproduced with permission from Ref. [35] (Copyright American Chemical Society, 2016).

The assembly of conformationally flat cyclic peptides approach has been later thoroughly developed by Granja et al., with recently reported and elegant examples [32,33]. Although several of the enantiopure structures proposed lose even the low *C_2_* symmetry of the prototypical system developed by Ghadiri, the chirality properties of the resulting chiral nanotubes have not been exploited to date. One of the most intriguing nanotubular system developed with cyclic peptides have been recently reported by Danial, Catrouillet et al. They combined self-assembling cyclic peptides with a pH-responsive polymer [34,35], creating pH-sensitive materials which can be used in pharmaceutical applications, such as drug delivery agents or membrane channels. The new structure is composed by a polymer, poly(dimethylamino ethyl methacrylate) (pDMAEMA), conjugated to a cyclopeptide (structure **1** in Figure 2). The cyclic peptide consists of alternating *D*- and *L*-amino acids with the sequence *L*-Lys-*D*-Leu-*L*-Trp-*D*-Leu-*L*-Lys-*D*-Leu-*L*-Trp-*D*-Leu, modified with two diametrically opposed pendant chains that are attached to the free amine groups of the lysine residues, and polymer chains are grown from the modified CP via the reversible addition-fragmentation chain transfer (RAFT) process. The system can be seen as intrinsically formed by diastereoisomers, since the statistically unequal length of polymer chains on the two diametrically opposite lysine residues breaks the local molecular *C*_2_ symmetry.

The self-assembly process could be studied in aqueous solution using Small-Angle Neutron Scattering (SANS), in order to monitor the effect of protonation on the morphology and the size of the aggregates and controlled by variation of pH; the assembly was fully reversible. Due to the basic tertiary amine groups on the polymer, pH = 9 is recorded when the (CP)-polymer conjugate is solubilized in D_2_O, leading to NTs with a number of aggregation of 15. When the solution is acidified by addition of small amounts of DCl, a decrease in the number of aggregation, to 8 at pH 8, 5 at pH 7, and finally 1 at pH 2, is observed. In acidic conditions, the conjugates become highly charged, causing an electrostatic repulsion between them, due to the protonation of the tertiary amine groups on the polymer, disrupting the self-assembly. To demonstrate the reversibility of the self-assembly process, the addition of NaOD restored the number of aggregation to 13 at pH 9.7.

**Figure 3:**
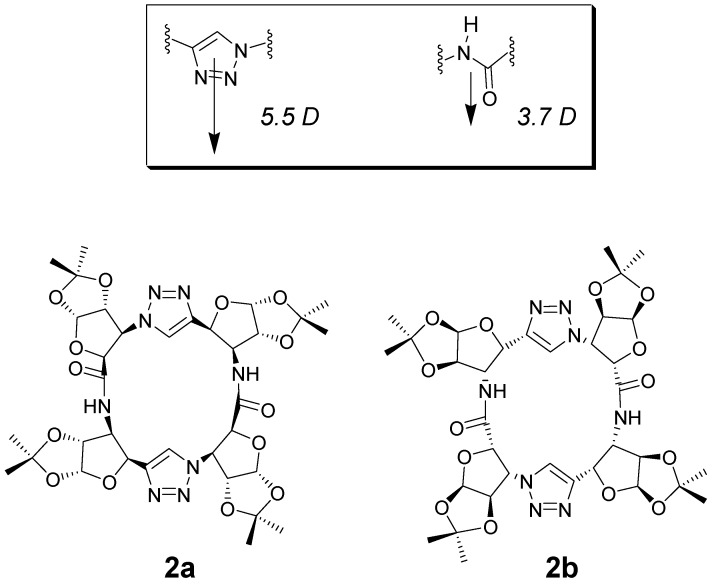
Comparison between triazole and amide functionalities, and diastereoisomeric rotamers of macrocycle 2. For the comparison between dipole moments, see Ref. [37].

More recently, a chiral 1,4-linked triazole/amide based peptidomimetic macrocycle has been reported by Ghorai et al. [36]. The synthesis of the molecular cyclic structure is based on the Cu-catalyzed alkyne-azide cycloaddition (CuAAC) reaction between alkyne and azide moieties of precursors, composed of *cis*-furanoid sugars and β-alanines as amino acids. Two regioisomeric chiral products **2a**,**b** (Figure 3) were separately obtained. The CuAAC reaction generates triazoles, which can be considered as amide bond analogues, in terms of planarity and polarity, as well as hydrogen bond donating and accepting ability. Nuclear Magnetic Resonance (NMR) and molecular modeling studies established that the conformation of the novel peptidomimetic macrocycle, essentially planar, is similar to that of (*D*,*L*)-α-amino acid based achiral cyclic peptides. The mode of self-assembly is however different from previous cases; in fact, the formation of a well-defined tubular chiral nanostructure has occurred, involving amide NH and triazole N2/N3 as well as by parallel stacking via amide NH and amide carbonyl oxygen H–bonding.

The concept of directionality in the assembly of enantiopure cyclic peptides have been pushed forward and addressed, with the synthesis of macrocyclic urea/amide hybrids, by Hennig et al. The derived NTs function as functional, anion-selective membrane transporters in lipid bilayer membranes. The authors describe derivatives with varying side chains (aliphatic and aromatic) and were able to engineer conformations both with parallel and antiparallel carbonyl dipoles: “antiparallel” macrocycles that self-assemble into “antiparallel” NTs, similar to the above described cyclic peptides; and “parallel” macrocycles that self-assemble into “parallel” oriented NTs, in which the overall nanostructure is itself intrinsically chiral with strong macrodipoles. The overall unusual characteristics of parallel NTs call for a new transport mechanism, where macrodipole-potential interactions account for voltage sensitivity and anion-macrodipole interactions account for anion selectivity [38].

Peptides, as short as dipeptides, contain all the molecular information needed to form well-ordered structures at the nanoscale. Dipeptides have been demonstrated to be able to grow supramolecular (chiral) NTs. Furthermore, they have been used as templates for the internal growth of metallic nanowires by Reches et al. [39]. The same authors have recently reviewed the significant advancements in the field of peptide nanostructures in the last few years [40]. Other more complex peptide architectures have been elegantly shown to form (chiral) NTs, but the chirality has not been specifically addressed [41,42].

In an approach that has some analogy with the cyclic peptides, Gattusoet al. have published the synthesis of large cyclic oligosaccharides which assemble in the solid state to form nanotubular structures [43,44]. The authors have established a synthetic protocol for the high-yielding cyclization of a series of suitably protected disaccharide precursors, containing both a glycosyl donor and a glycosyl acceptor. After cyclization in diluted conditions in the presence of TrClO_4_ as the catalyst, and deprotection, a series of cyclic oligosaccharides have been obtained and characterized. In particular, cyclic oligosaccharidic octamers or decamers obtained by *L*-mannopyranose and *D*-mannopyranose containing monomers (MM), or *L*-rhamnopyranose and *D*-rhamnopyranose containing monomers (*RR*), are achiral, since they possess *S*_8_ or *S*_10_ molecular symmetry. X-ray crystallographic investigation demonstrates the presence of the described molecular symmetry, and the cyclic achiral octasaccharide and decasaccharide belonging to the *RR* series revealed infinite stacks to form nanotubes ranging from 1 nm for the cyclic octasaccharide to 1.3 nm for the cyclic decasaccharide in diameter. Nanotubular structures are held together by loose van der Waals contacts.

**Figure 4:**
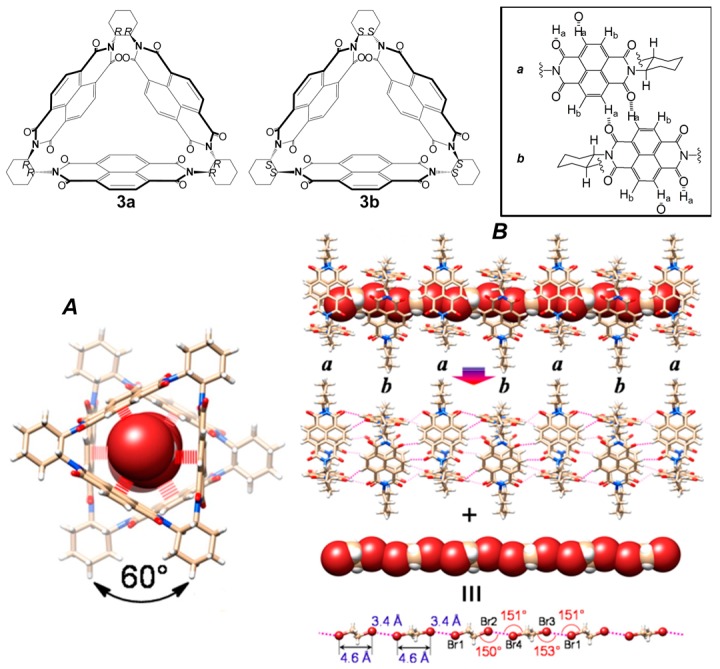
*R*-Δ and *S*-Δ macrocycles **3a**,**b** (top left). Schematic view of the [C–H···O] interactions between the NDI units (top right): (**A**) bottom: top view showing that the coaxial DBA chain is stabilized through latitudinal [Br···*π*] interactions; and (**B**) bottom schematic illustration of nanotubular structure. Partially reproduced with permission from Ref. [45] (Copyright American Chemical Society, 2014).

A very interesting approach to create chiral NTs is the guest-assisted assembly of macrocycles. Liu et al. reported the specific assembly of supramolecular NTs, starting from a pair of enantiomeric rigid triangular naphthalenediimide-based macrocycles with tubular cavities, namely (*RRRRRR*)- and (*SSSSSS*)-NDI-Δ (*R*-Δ and *S*-Δ, respectively), used as hosts, and a class of solvents, namely 1,2-dihalohydrocarbons, used as guests [45]. The macrocycles **3a**,**b** (Figure 4) were synthesized combining three naphthalenediimides (NDIs) with three (*RR*)- or (*SS*)-*trans*-1,2-cyclohexanediamine units as linkers.

Solvents including 1,2-dihalo-ethanes and -ethenes (DXEs) are necessary to create host-based tubular structures, as a result of a cooperation between the [halogen···halogen] interactions and the weak hydrogen bonds among NDI-Δ units, since the latter are not sufficient to hold them together in a columnar manner to produce NTs. The authors demonstrated that, depending on the solvent, different supramolecular structures can be formed. Using the *R*-Δ macrocycle and (*E*)-1,2-dichloroethene ((*E*)-DCE), an organogel immediately formed, probably due to the electron-rich C=C double bond and its *anti*-conformation. The gel presented a composition of nanofibers, revealed by SEM and AFM microscopies. X-ray diffraction (XRD) analysis showed the formation of tubular nanostructures. Single-crystal X-ray superstructure of DBA&*R*-Δ, composed by *R*-Δ macrocycle and BrCH_2_CH_2_Br (DBA), presented a nonhelical tubular structure, showing the coaxial stacking of two *R*-Δ molecules *a* and *b* involving three pairs of [C–H···O] interactions between the NDI units, with a perpendicular rotation angle of 60° (Figure 4). The *R*-Δ *ab* pairs constitute a pattern which supports a hexagonal channel, perfectly filled and stabilized by a [Br···Br]-bonded DBA chain, with latitudinal [Br···*π*] interactions between the Br atoms of the bridging DBA molecules and the NDI planes of *R*-Δ *ab* pairs. The DBA chain is composed of two kinds of DBA molecules, that adopt *anti* conformations and are arranged in an alternating manner linked by longitudinal [Br···Br] bonding interactions (Figure 4). The same mechanism is observed for the single-crystals X-ray superstructures composed by *R*-Δ macrocycle and ClCH_2_CH_2_I (CIA).

**Figure 5:**
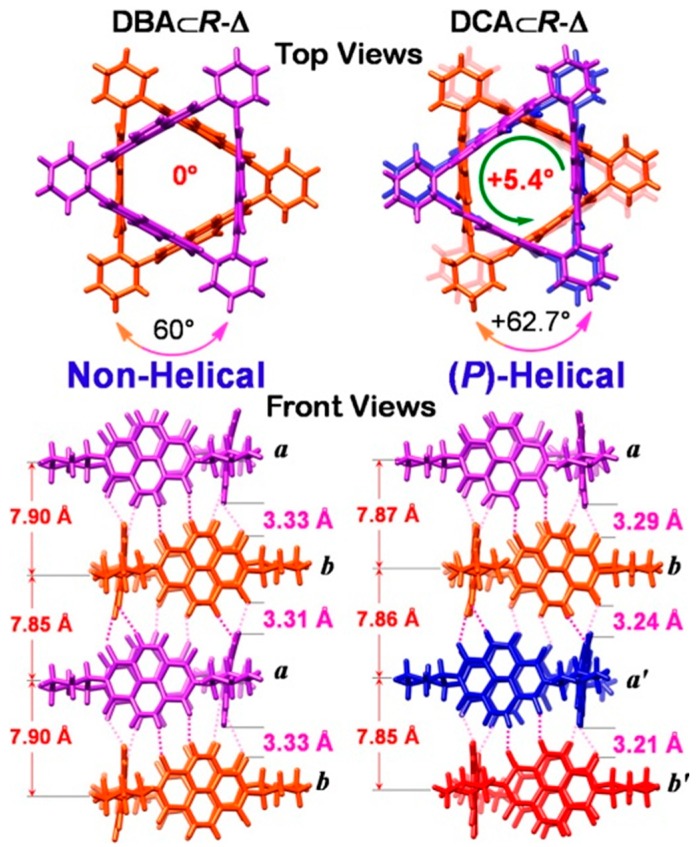
Comparison of single-crystal X-ray superstructures: nonhelical tetrameric unit of DBA&*R*-Δ (on left) and (*P*)-helical tetrameric DCA&*R*-Δ (on right). Reproduced with permission from Ref. [45] (Copyright American Chemical Society, 2014).

In contrast with DBA&*R*-Δ, the *R*-Δ macrocycle and ClCH_2_CH_2_Cl (DCA) formed a tetrameric, single-handed (*P*)-helical nanotubular structure, showing the coaxial stacking of four *R*-Δ molecules *a*, *b*, *a’* and *b’* involving nine interfacial pairs of [C–H···O] interactions, with a counterclockwise rotation angle of +62.7°, rather than 60°. The axes of the tetramer do not overlap completely but exhibit net counterclockwise rotation angles of +5.4°. The enantiomeric DCA&*S*-Δ showed the single-handed (*M*)-helical structure. The comparison between DBA&*R*-Δ and DCA&*R*-Δ is shown in Figure 5.

**Figure 6:**
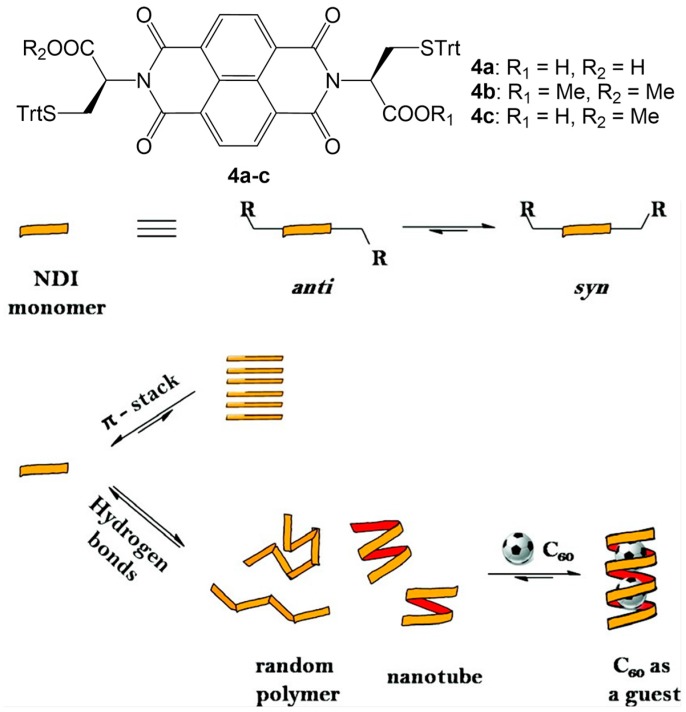
Amino acid functionalized NDIs **4a**–**c**. Schematic representation of the equilibria between *anti* and *syn* conformations of the NDI monomer (on top) and possible NDI aggregates (above). Partially reproduced with permission from Ref. [46] (© American Chemical Society).

Ponnuswamy et al. reported a class of amino acid functionalized NDIs, able to aggregate into hydrogen-bonded helical NTs. They could investigate NDI aggregates in solution using *S*-trityl protected, cysteine functionalized NDIs **4a**–**c** (Figure 6), due to their good solubility in chlorinated solvents [46].

The self-assembly of the NDI monomers can occur via *π*-stacking and hydrogen bonds. In aprotic solvents of medium polarity, such as CHCl_3_, the formation of *π*-stacked polymers is unfavorable, due to the electron deficient character of the NDI aromatic core and the steric hindrance of the amino acid side chains. Considering the two possible *anti* or *syn* conformations which can be engaged by the side chains of the NDI monomers, supramolecular polymers via hydrogen bonds formation can be formed. Both NDI conformers can self-assemble via COOH dimerization to produce random polymers, but only the poly-*sin*-NDI is able to form supramolecular NTs (Figure 6).

**Figure 7:**
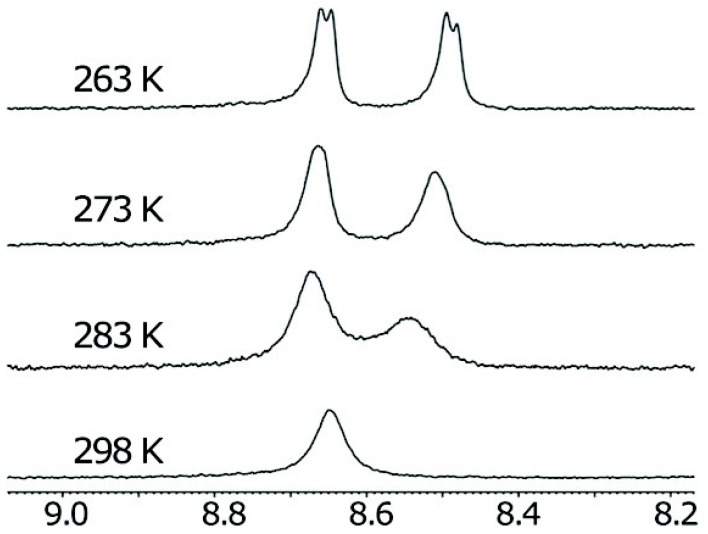
^1^H-NMR spectrum of the NDI protons of a solution of **4a** in TCE, at different temperatures. Partially reproduced with permission from Ref. [46] (Copyright American Chemical Society, 2012).

The supramolecular polymerization of these NDI helical NTs occurs via a non-cooperative, isodesmic process, in which the addition of each monomer to the growing chain is governed by a single equilibrium association constant. In order to investigate the thermodynamics of the formation of the NTs, temperature- and concentration-dependent proton NMR (NMR) experiments were performed on NDIs **4a**–**c** in CHCl_3_ or 1,1,2,2-tetrachloroethane (TCE): **4b** cannot form hydrogen bonds and **4c** can only form a hydrogen-bonded dimer, while the monomer **4a** demonstrated a highly dependence on both concentration and temperature. The ^1^H-NMR spectra revealed that, in a random hydrogen-bonded polymer, the NDI aromatic protons show a symmetric environment, appearing as a broad singlet; contrarily, in the NT, at lower temperatures, a splitting of the NDI peak into two peaks can be observed, supporting the formation of a well-defined hydrogen-bonded supramolecular structure with two different environments for the NDI protons (Figure 7).

^1^H-NMR experiments recorded at different temperatures and concentrations of **4a** in CHCl_3_ suggested that the formation of the NT is enthalpy driven and entropically disfavoured, because of the restriction of the conformational freedom due to the hydrogen bonds between NDI monomers. Furthermore, the degree of polymerization showed a greater number for a solution of **4a** in TCE at 273 K, respect to 300 K, revealing the presence of NTs up to 9 helical turns, corresponding to 28 aggregated NDI monomers. Association studies could also be performed using concentration-dependent circular dichroism (CD) spectroscopy. The solvation and the introduction of guests can change the stability of the NTs: the addition of a noncompetitive solvent, such as cyclohexane [47], to a solution of **4a** in TCE showed an increase of the strength of hydrogen bonds in the NDI NT; the encapsulation of C_60_ in the tubular structure (Figure 6) allows its stabilization, due to the good match between the solvophobic surface of guest and the inner walls of the host NT, increasing degree of polymerization. In addition to C_60_, NDI NTs can also act as receptors for ammonium ions in a solution of CHCl_3_: an interesting competition experiment with C_60_ and ammonium ions revealed the formation of mixed complexes, with both guest molecules present in the cavity of NT.

As control experiments, the authors modified the side chains of the NDI monomers, introducing *ε*-NHBoc protected lysine **5** and isoleucine **6** functionalized NDIs, and *N*-desymmetrized NDIs, whereby **7** and **8**, with a *S*-trityl protected cysteine on one side and respectively, *ε*-NHBoc protected lysine or *ε*-NH-thiourea lysine, on the other. The association constants and CD studies revealed a formation of less stable NTs of molecules **5** and **6**, compared to **4a**, due to: (i) solvophobic effects; (ii) competition for COOH groups to form hydrogen-bonded dimers with amide groups present in the Boc side chain of **5**; and (iii) the higher steric repulsion caused by the β-substituent in **6**. Furthermore, in the case of compound **8** compared to **7**, thiourea gives an additional stabilization of the helical NT through non-covalent hydrogen-bonded cross-linking; however, the presence of the thiourea compensates the loss of the trityl group, which has a stabilizing effect via solvophobic interaction, revealing the same stability of the NTs of **8** and **4a**.

Another recent example of guest-assisted host assembly of small molecules was reported by Shi et al., in which a stimuli-responsive H-bonding monomer changes its aggregation mode in response to different solvents or in the absence and presence of C_60_ and C_70_ guests [48]. 

**Figure 8:**
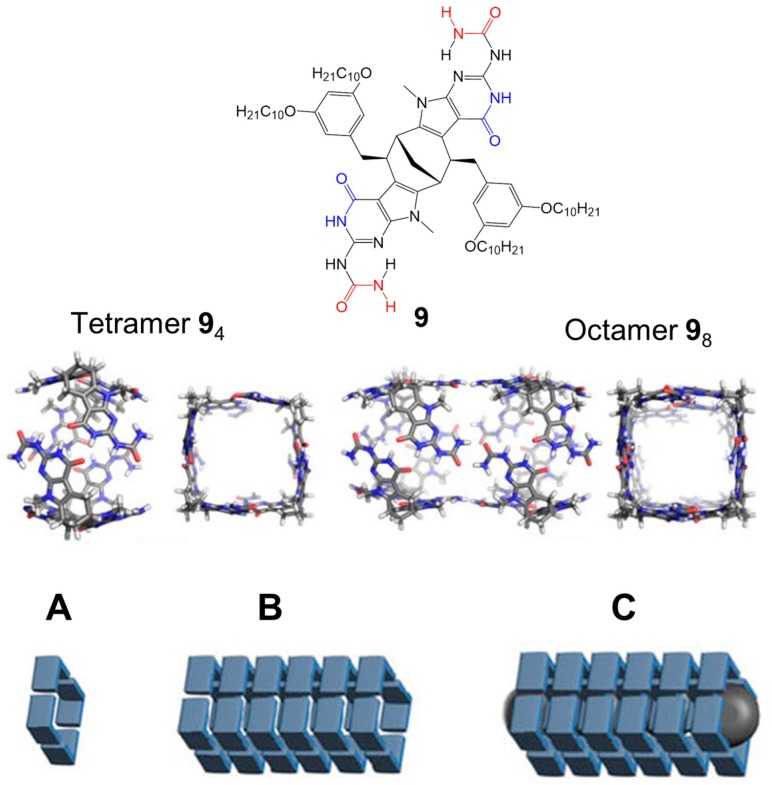
Monomer **9** with two orthogonal 2H-bonding sites (marked as red and blue). Molecular models of 2H-bonded tetramer **9**_4_ and the corresponding polymeric tube, represented as **9**_8_. Schematic representation of: (**A**) tetrameric cyclic aggregate in CDCl_3_; (**B**) nanotubular structure in toluene-*d*_8_; and (**C**) nanotubular structure in CDCl_3_ with C_70_ as guest. Partially reproduced with permission from Ref. [48] (Copyright Nature Publishing Group, 2017).

The novel enantiopure bicyclic compound **9** (Figure 8) presents a bicyclo[3.3.1]nonane (BCN) core symmetrically fused with two *N*-methyl pyrrolopyrimidin-4-one moieties, bearing unsubstituted ureas. The intra-cyclic H-bonding site (marked as blue in Figure 8) might be expected to be used for the connection of the monomers into a cyclic tetramer while the terminal urea units (marked as red in Figure 8) might be the sites to link of the resulting noncovalent macrocycle into a 2H-bonded polymeric tube. ^1^H-NMR and gel permeation chromatography (GPC) analysis showed that the monomeric compound exists as a single H-bonded tetrameric cyclic aggregate (Figure 8A) in CHCl_3_, with a polydispersity index of 1.02. A possible reason for the formation of the tetramer is due to the weak H-bonds between terminal urea units, in contrast to the strong intra-cyclic H-bonds. Thus, in order to increase the strength of the H-bonds, ^1^H-NMR experiments were performed in aromatic solvent, such as toluene and benzene, revealing the formation of high molecular-weight aggregates, composed by 15–20 cyclic tetrameric units in the chain (Figure 8B); GPC analysis detected a bimodal distribution, probably due to a mixture of oligomers and cyclic tetramers, which disappeared during ageing, displaying a single peak corresponding to the supramolecular polymer. The formation of the polymeric tubular structures was also detected with the addition of a C_70_ guest to a solution of the monomer **9** in CDCl_3_ (Figure 8C): this mechanism is unknown but ultraviolet-visible studies showed that the guest is incorporated into the cavity of the tube, since the absorption band of C_70_ is shifted bathochromically.

**Figure 9:**
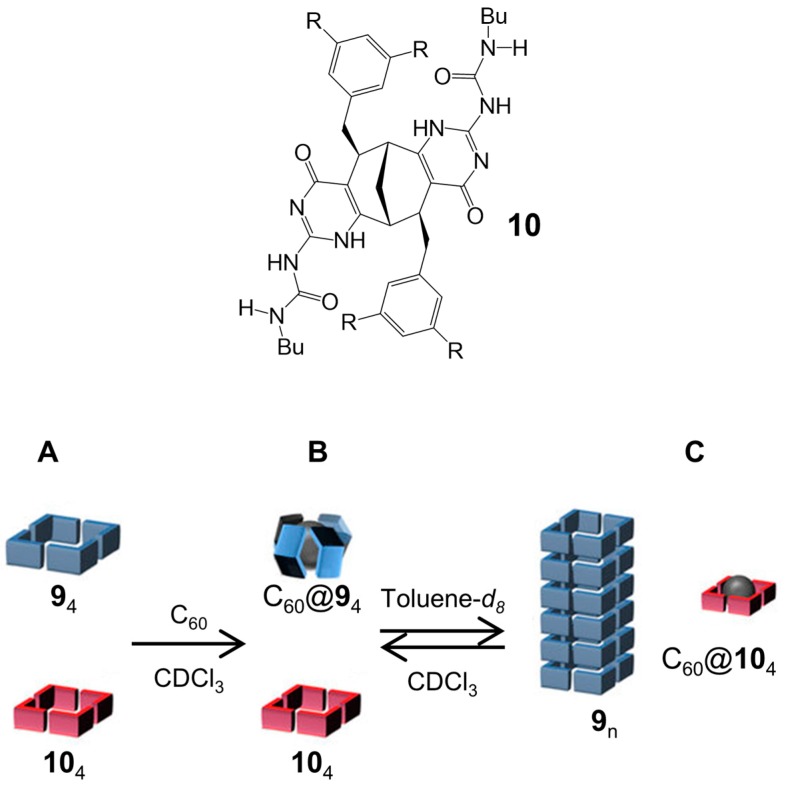
Monomer **10**; schematic representation of: (**A**) tetrameric cyclic aggregates in CDCl_3_; (**B**) capsule-like insertion complex C_60_@**9**_4_ and tetramer **10**_4_, in CDCl_3_; and (**C**) capsule-like insertion complex C_60_@**10**_4_ and nanotubular structure **9**_n_, in toluene-*d_8_*. Partially reproduced with permission from Ref. [48] Copyright Nature Publishing Group, 2017).

The addition of a C_60_ guest to a solution of the monomer **9** in CDCl_3_, in contrast to the polymeric aggregates formed with C_70_, highlighted the formation of a well-defined inclusion complex, in which two different conformers were present: one conformer has an intramolecular H-bond between a N–H proton of the isocytosine and one of the urea C=O groups while the other conformer has an intramolecular H-bond between a urea N–H donor and an isocytosine acceptor. In order to demonstrate the role of C_60_ as a selective switch in a multicomponent supramolecular system, a second bicyclic compound **10** was considered (Figure 9). Compound **10** forms a cyclic tetrameric aggregate in CDCl_3_, which does not interact with C_60_ guest. In the presence of both **9** and **10** (1:1 mixture) in CDCl_3_ solution, ^1^H-NMR experiments revealed that no exchange of monomers between two tetramers **9**_4_ and **10**_4_ took place (Figure 9A). The addition of a C_60_ guest to the solution resulted in the selective formation of a capsule-like insertion complex C_60_@**9**_4_, leaving **10**_4_ intact (Figure 9B). The replacement of CDCl_3_ with toluene-*d_8_* created the rearrangement of the complex C_60_@**9**_4_ into the tubular polymer **9**_n_ with the contemporary insertion of C_60_ into the cavity of the tetramer **10**_4_: this process has been demonstrated to be reversible (Figure 9C).

### 2.2. *π*–*π* Stacking Interactions

The *π*–*π* stacking of aromatic compounds is a type of noncovalent interaction widely used in supramolecular chemistry for construction of nano objects. *π*–*π* Stacking was first thoroughly examined by Sanders and Hunter in the early 1990s [49].

**Figure 10:**
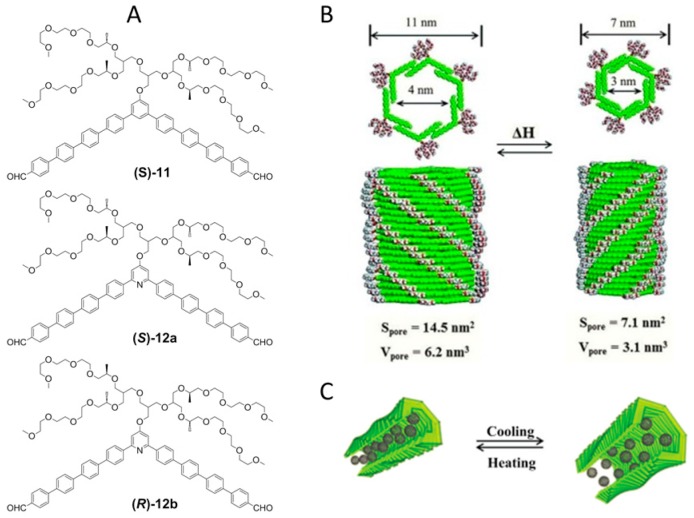
(**A**) Building blocks used in self-assembly of noncovalent NTs; (**B**) NTs section formed from **12a** with reversible dilatation-contraction in function of thermal response; and (**C**) stimuli response on NTs in function of the temperature in sensing of C_60_. Reproduced with permission from Ref. [50] (Copyright the American Association for the Advancement of Science, 2012).

Huang et al. in a recent approach [50] build on previous achievements dealing with NTs obtained through *π*–*π* stacking interaction between covalent macrocycles, by assembling noncovalent aromatic macrocycles (**11**, **12a** or **12b**) in which adjacent aromatic segments interact with one another by *π*–*π* stacking interactions, defining the macrocyclic structure. The noncovalent macrocycles spontaneously stack on top of each other in diluted aqueous solution to form a tubular structure with a hydrophobic interior. The self-assembling molecules that form this aggregate consist of a bent-shaped aromatic segment with an internal angle of 120° and with aldehyde moiety as the terminal group. The chirality of the correspondent NTs is guaranteed by the design of the hydrophilic oligoether dendron grafted at its apex, in which the side chains possess chiral centers of the (*S*) configuration (compounds **11** and **12a**) or the (*R*) configuration (compound **12b**) (Figure 10).

The molecular weight of the primary aggregate, obtained by vapor pressure osmometry (VPO) measurements, resulted to be six times as large as that of the single building blocks indicating, together with inspection of Corey-Pauling-Koltun (CPK) models, that a single noncovalent macrocycle precursor of NTs is formed via fully overlapped packing arrangement of six unit of building blocks (Figure 10B). In NTs formed by self-assembly of **11**, TEM measurements showed elongated object with an external diameter of 7 nm and a hollow interior with a diameter 3 nm. *H*-type stacking occurs between the aromatic segments of monomeric **11** units, as observed by Ultraviolet-Visible (UV-Vis) and fluorescence spectroscopies. CD spectra in aqueous solution showed a significant Cotton effect, indicating that the tubes adopt a one-handed helical structure. Changing building blocks to **12a** and **12b** afforded important structural modifications in the correspondent NTs: the increment of external and internal cavity diameter observed by TEM imaging, and a *J*-type stacking of the aromatic segments, suggest an expansion of the noncovalent macrocycles. The introduction of a pyridine unit on the concave side of the apex of the bent shaped aromatic segment induce adjacent molecules to slide into a looser packing arrangement because pyridine is well-known to form water clusters through hydrogen bonding.

The innovative and very interesting aspect of these NTs is their capability of dynamic motion in response to external stimuli. The hollow tubes with oligoether dendritic exterior and pyridine interior exhibit thermoresponsive behavior due to thermally regulated dehydration capability of the ethylene oxide chains and the pyridine moieties. The reversible expansion-contraction motion of cavities of the NTs obtained from **12a** or **12b**, corresponding respectively to the hydrated and dehydrated forms, can be activated thermally by modifying the temperature of the aqueous solution from room temperature to 60 °C and vice versa. The dynamic motion of the tubules can be accompanied by variations of encapsulated hydrophobic guest molecules (Figure 10C). The NTs can take up C_60_ molecules through hydrophobic interactions during assembly. Authors have observed that upon addition of C_60_ to a solution of **12a**, the fluorescence intensity was considerably suppressed up to a maximum of 0.8 equivalent of C_60_, indicating that C_60_ was effectively encapsulated within the hydrophobic interior of the tubules at maximum value of 0.8 equivalent. On heating, a portion of the C_60_ guest population was released from the tubular interior due to the shrinkage of the tubes. The absorbance and solution color immediately recover to those of the contracted state upon subsequent heating, indicating that the breathing motion of the tubes leads to a reversible switch between tight and loose packing of the C_60_ moieties.

**Figure 11:**
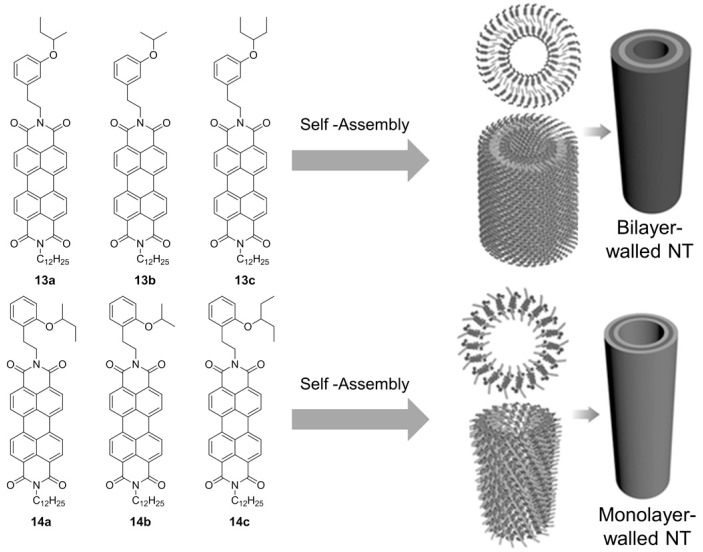
PDI-based building blocks for construction of chiral NTs. Partially reproduced with permission from Ref. [52] (Copyright Wiley-VCH, 2016).

CD-active NTs via *π*–*π* stacking interaction can be achieved by supercoiling of nanohelices as described by Ma, Peng et al. [51,52] using a family perylene diimide (PDI) molecules as building blocks. The NTs have tunable diameters and wall thicknesses via simple molecular engineering, from two new classes of asymmetric PDIs compounds. The molecular design developed includes a perylene diimide core linked through the nitrogen atoms to a linear C12 chain and, by an ethylene bridge, to a bulky, branched *m*- or *p*-substituted alkoxyphenyl moiety (Figure 11). Design studies conducted have suggested the crucial rule of the ethylene linkage to provide the necessary flexibility required for the formation of the nanotubular structure. Molecules **13a**–**c** form bilayer-walled nanotubes, whereas molecules **14a–c** form monolayer-walled NTs featuring an alternative geometry of the side chains within the *π*-stacking of the PDI molecules (Figure 11). More interestingly, the diameter of the bilayer NT dilates or contracts by changing the size of the branched substituents at the meta-position of the phenyl moiety of **13**. In fact, nanotubes obtained from **13c** exhibit a uniform external diameter of ≈25 nm, whereas the nanotubes obtained from **13a** and **13b** show uniform external diameters of ≈17 and ≈14 nm, respectively. This ability to change the external diameter with the size of the branched substituents is not observed in monolayer-walled NTs formed by **14a**–**c**. The use of racemic compounds **13a**–**c** and **14a**–**c** leads to CD silent NTs, but when enantiomeric pure (*R*)- or (*S*)-**13a** were used, a CD signal in the range of 460–660 nm is observed, indicative of the formation of single-handed NTs. All NTs exhibited high fluorescence quantum yields exceeding 43% due to their perylene moieties, with the monolayer-walled NTs being the less photostable. Because of their combination of tunable diameter, interior nanoporosity, robust photostability, and high fluorescence, these bilayer-walled NTs are ideal for applications as fluorescent sensors.

**Figure 12:**
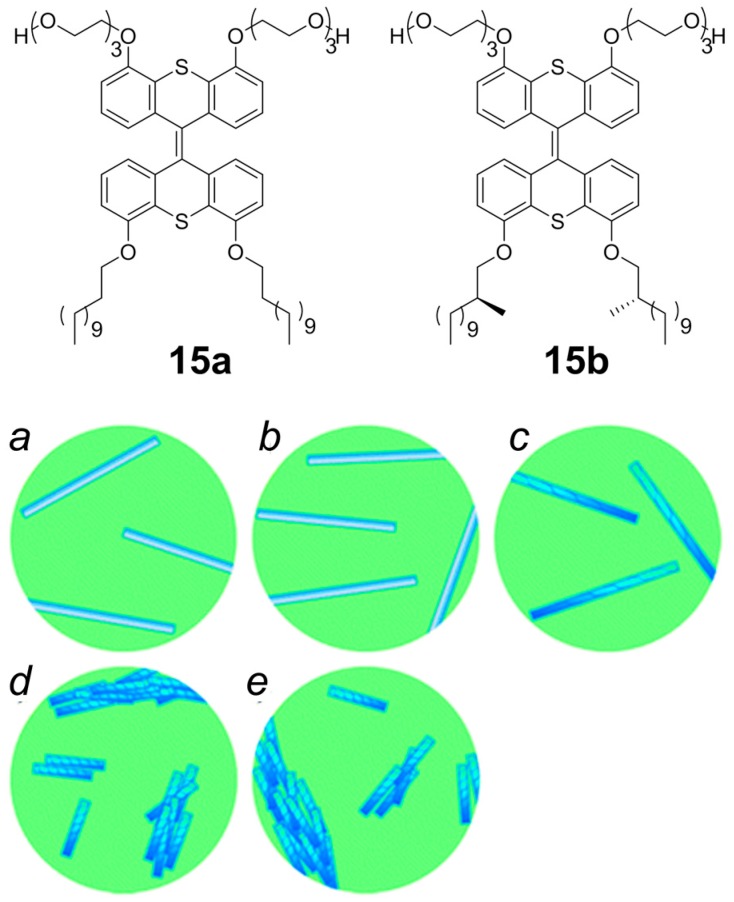
Top: Structure of amphiphile **15a** and **15b**. Bottom: Schematic model for the observed behavior of NTs consisting of **15a** and **15b** as a function of the amount of **15b**: (**a**) pure **15a**; long, isolated achiral nanotubes; (**b**) <25% **15b**; long, isolated achiral nanotubes; (**c**) 25–50% **15b**; long, isolated chiral nanotubes; (**d**) >50% **15b**; short, bundled chiral nanotubes; and (**e**) pure **15b**; short, bundled chiral nanotubes. Partially reproduced with permission from Ref. [53] (Copyright Royal Society of Chemistry, 2017).

Van Dijken et al. [53] have reported the synthesis of enantiomerically pure supercoiled helical NTs in water simply doping the achiral tubular structure of amphiphile **15a** with small amounts of a closely related enantiopure analogue **15b**. Only small amounts of enantiomerically pure compound is required to control CD-response and, more importantly, the morphology of the chiral NTs (Figure 12). The structures of amphiphiles **15a** and **15b** contain a photosensitive overcrowded alkene unit that links two hydrophilic oligo-ethylene glycol headgroups with two hydrophobic alkyl tails. The capability of photoisomerization of the bis-thioxanthylidene core when exposed to light provides a facile NTs disassembly methodology, and the oligo-ethylene glycol units facilitate its solubility in water. The hydrophobic alkyl chains are positioned at the core structure in order to maximize interaction and to facilitate supramolecular assembly. Chiral information in compound **15b** is brought by two stereocenters in the hydrophobic chains. Distinct differences in morphology between the NTs of pure achiral **15a** and chiral **15b** were observed by cryo-TEM. NTs of **15a** (Figure 12a) are typically longer than a micrometer, very straight and generally isolated amongst them, while the tubes of **15b** (Figure 12e) are shorter (typically only ≈300 nm), and tend to aggregate. CD response of chiral NTs formed from **15b** exhibits Cotton effect, whereas as expected NTs formed from **15a** are CD silent. Having confirmed that both **15a** and **15b** form NTs, the author have studied the effect of doping the achiral NTs, with fixed amount of **15b**, performing sergeant-soldiers experiments. The mixed NTs formation was not inhibited at any ratio of the amphiphile **15a**:**15b**. NTs with less than 25% of **15b** do not show any CD signal and in Cryo-TEM imaging the NTs appeared with morphology detected for achiral NTs of **15a** (Figure 12b). With a ratio of amphiphile **15a**:**15b** between 25–50% of **15b** a CD signal was recorded, and Cryo-TEM imaging showed that mixtures NTs are micrometers long and with tendency to be straight until **15b** became the major component (>50%) (Figure 12c). When the fraction of **15b** is over 50%, CD signal intensity tends to a plateau and Cryo-TEM imaging showed the typical morphology of chiral NTs (Figure 12d). These results show that chirality is a distinctive factor in controlling the length of individual self-assembled NTs, the aggregation of NTs and the chirality of the assembly.

### 2.3. Metal-Organic Interactions

Metal-organic interactions have been attracting considerable attention from chemists and material scientists in the formation of 3D metal-organic frameworks (MOFs) due to their great potential in the areas of gas adsorption and energy storage. Elements of chirality have already been introduced into MOFs [54,55,56,57,58,59]. In the assembly of chiral NTs, it is especially important to carefully choose organic ligands and metal ions, search crystallization conditions, including guest molecules, and adopt appropriate synthesis strategies.

**Figure 13:**
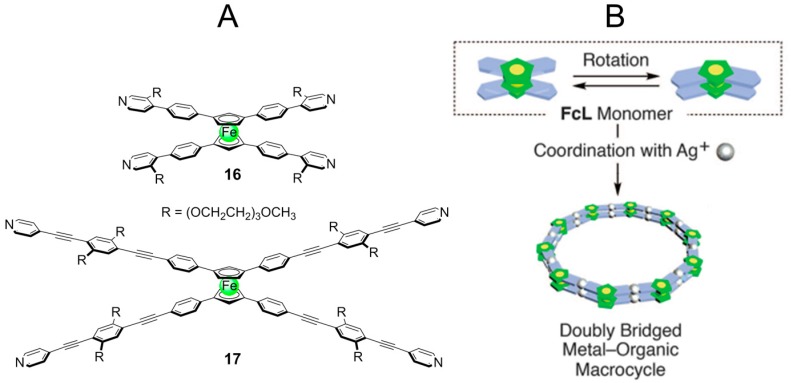
(**A**) Building blocks used for construction of NTs; and (**B**) schematic illustration of possible noncovalent structure upon complexation with Ag(I). Partially reproduced with permission from Ref. [60] (Copyright the American Association for the Advancement of Science, 2014).

Fukino et al. [60] reported the noncovalent synthesis of large-diameter NTs, which can be preferentially “cut” into its building blocks. A key for the success in cutting the NTs is the redox-active ferrocene motif used in metal-ligating units **16** and **17** (Figure 13).

**Figure 14:**
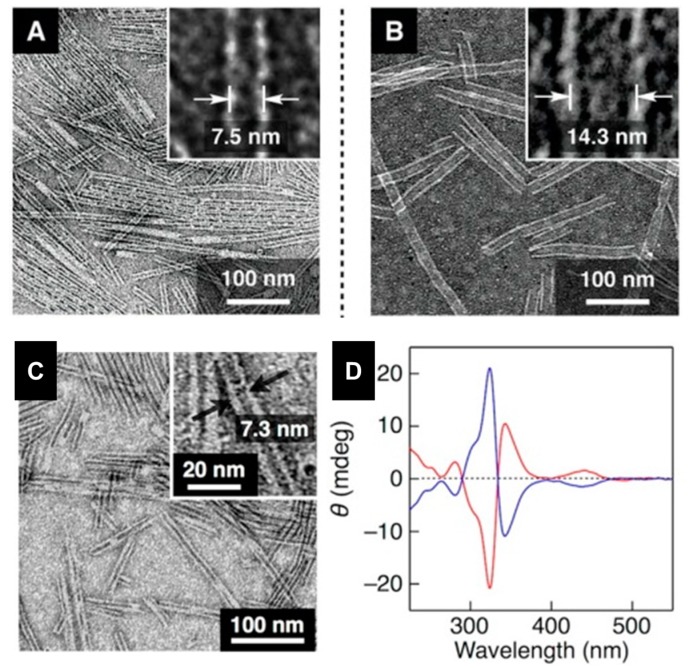
TEM micrographs of air-dried MeCN/water dispersions of: (**A**) **16**; (**B**) **17**; (**C**) CD active NT formed by **16** in presence of (+/−)-MS; and (**D**) CD spectrum of NTs obtained using (+)-MS (red line) and (−)-MS (blue line). Partially reproduced with permission from Ref. [60] (Copyright the American Association for the Advancement of Science, 2014) and [61] (Copyright the American Chemical Society, 2015).

The tetratopic ligands carry a metal-coordinating 4-pyridyl group at each terminus of the four aromatic arms. Triethylene glycol (TEG) side chains are introduced to enhance solubility of the ligands and to provide better dispersibilities of assembled products in common organic solvents. As a metal ion source, AgBF_4_ was chosen because of its well established capability of coordination with pyridine derivatives. NTs obtained in MeCN were 50 nm to 2 μm long and show a uniform diameter of 7.5 nm and 14.3 nm respectively for **16** and **17** (Figure 14A,B). Taking into account the bent angle of **16** and **17** (144°), these NTs diameter allows for the construction of a molecular model with a decagonal cross section (vertex angle; 144°) composed of 10 bent-shaped ligands and 20 Ag(I) ions. The intrinsically achiral structure of building blocks did not lead to formation of chiral NT. However, a closer examination of the 2D X-ray diffraction (2D XRD) image of the magnetically oriented NTs indicated that a small helical twist may exist in the stacking geometry. If this helical twist can unidirectionally be developed over the entire nanotube, NTs would be optically active.

As described by the same authors [61], when the coassembly was carried out in the presence of (*+*)- or (*−*)-menthylsulfate (MS*−), the resultant NTs indeed displayed an optical activity exhibiting Cotton effects at 325 nm and 343 nm in its CD spectrum (Figure 14C,D).

**Figure 15:**
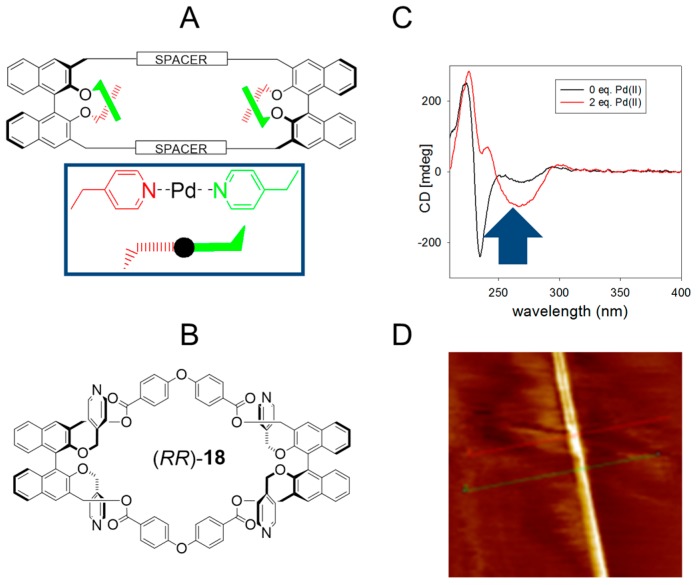
(**A**) Cartoon representation of the design; (**B**) chemical structure of the *D*_2_ symmetrical macrocycle (*RR*)-**18**; (**C**) CD spectra of the macrocycle, and of the aggregates in MeCN solutions upon addition of two equivalents of the bidentate Pd^2+^; and (**D**) AFM images of fibers formed by (*RR*)-**18**, C60 and PdCl_2_(MeCN)_2_, with cross section of ca. 1 nm. Partially reproduced with permission from Ref. [62] (Copyright Royal Society of Chemistry, 2015).

Caricato et al. [62] recently reported the metal-organic induced nanoscale organization of a BINOL-based macrocycle. This report continues a series of contributions from the same group, on chiral nanostructuring and chiroptical sensing [63,64,65,66,67,68,69,70,71,72]. The innovation in the design resides in the incorporation of a robust, chromophoric source of axial chirality into a cyclic, shape persistent covalent framework (Figure 15A). The homochiral macrocycle (*RR*)-**18** possesses an overall molecular *D*_2_ symmetry, and multivalency is introduced into the covalent framework by means of four suitably positioned pyridine moieties. Upon addition of Pd^2+^, coordination of the pyridine moieties occurs both intra- and intermolecularly, to afford chiral ordered mono and dimeric macrocycles or multimeric aggregates depending on the solvents and conditions used. The metal binding event takes place in combination with a significant macrocyclic conformational rearrangement detected by CD spectroscopy (Figure 15C). When in combination with a third component (C_60_), the macrocycle-Pd^2+^ hybrid undergoes surface-confined nanostructuring into chiral NTs, with extremely high aspect ratio (cross section of ca. 1 nm, Figure 15D).

**Figure 16:**
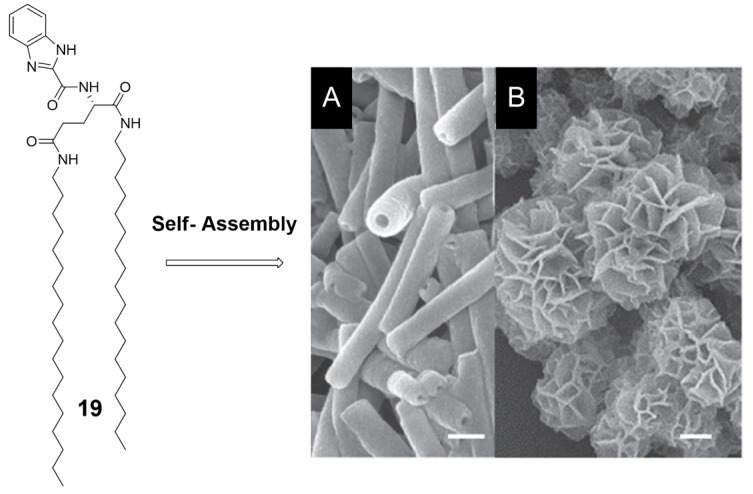
Molecular structure of **19** and fabrication of hierarchical chiral nanostructures, which was based on the self-assembly of **3** and regulated by solvents and metal ions: (**A**) nanotubes (scale bar = 100 nm); and (**B**) flower-like structures (scale bar = 1 μm). Partially reproduced with permission from Ref. [73] (Copyright Wiley-VCH, 2016).

Zhou et al. [73] designed low-molecular-weight organogelator **19** containing a benzimidazole moiety linked an amphiphilic *L*-glutamic amide, able to form chiral nanotubes in polar solvents (DMF and acetone) (Figure 16). The enantiopure amphiphilic glutamide part was used as the gelator moiety, while the heteroaromatic benzimidazole moiety was introduced for *π*–*π* stacking, H-bonding and metal coordination. NTs were obtained in gel phase, confirmed by TEM characterization and shown in Figure 16A (hollow outer diameter of 85–100 nm, hollow inner diameter 34–40 nm, wall thickness of 25–30 nm), with CD spectroscopy showing a Cotton effect in DMF and acetone in the chromophore region. The well-known capability of benzimidazole to coordinate lanthanide ions was used by the authors to promote the formations of 3D tubular motif (Figure 16B). The authors have observed, upon addition of Eu(NO_3_)_3_ and Tb(NO_3_)_3_, the formation of tuber flower structures, whose outer diameter ranged from 50 to 80 nm as measured from SEM, which were hierarchically constructed by a large number of NTs.

### 2.4. Multiple Interactions

**Figure 17:**
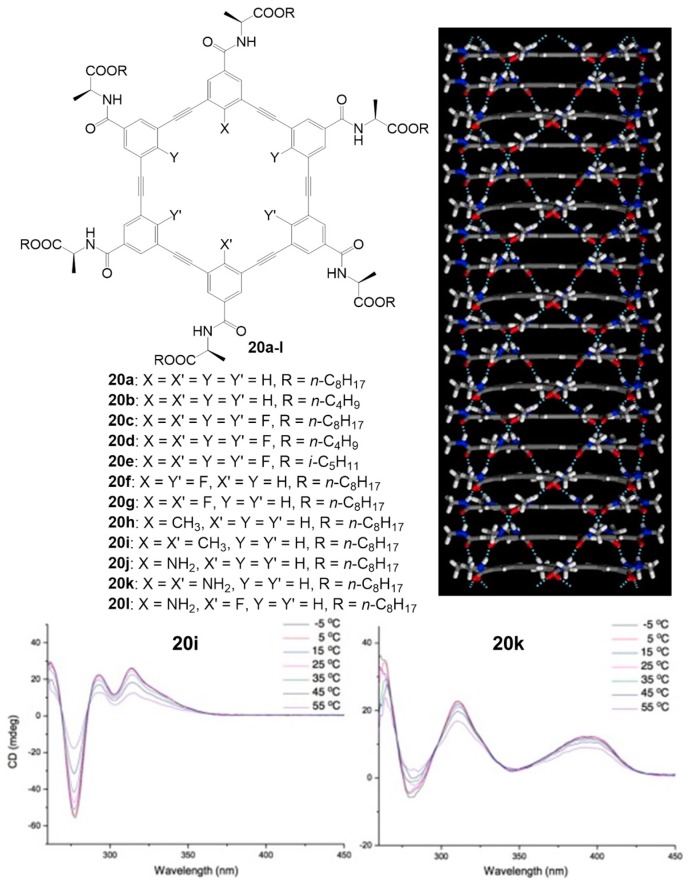
Structures of *m*-PE macrocycles **20a**–**l** (top left), QMD simulation of a helical stack of the macrocycles (top right), and variable-temperature CD spectra of **20i** and **20k** measured in CCl_4_ (10 µM) (bottom). Partially reproduced with permission from Ref. [80] (Copyright Nature Publishing Group, 2012) and [81] (Copyright the American Chemical Society, 2016).

Nanotubes can be also formed by multiple interactions, such as the cooperation of multiple hydrogen bonding and *π*–*π* stacking interactions. Tubular structures made by self-assembly from rigid macrocycles containing oligo-(phenylene ethynylene) backbones have been pioneered by the groups led by Moore et al., Schlüter et al. [74,75,76,77,78,79], and the assembly made to order essentially by *π*–*π* stacking between the large cyclic aromatic surfaces. A new class of hexakis(*m*-phenylene ethynylene) macrocycles has been recently reported by Zhou et al. [80]. The stability of such nanotubes is connected to the presence of multiple hydrogen-bonding side chains further to *π*–*π* stacking involving the backbones. The modulation of the stacking strength of the helical nanotubular assemblies depends on the presence of different functional groups in the inner cavities of macrocycles [81]. Compounds **20a**–**l** (Figure 17) were prepared following a convergent synthetic sequence that involves a Pd-catalyzed Sonogashira coupling of monomeric building blocks to give trimeric precursors, which are recombined in a ring-closure reaction to give macrocycles.

The internal functional groups influence the stacking propensity of the resultant macrocycles: electron-withdrawing groups decrease the electron density of the backbone and increase the *π*–*π* stacking, while electron-donating groups enhance electron density decreasing the stacking. A study was performed by comparing compounds **20g**, **20i**, and **20k**, which contain two functional groups in their cavity, and **20c**, with six fluorine atoms in its cavity, with **20a**. The ^1^H-NMR signals of these macrocycles are broadened in spectra recorded in CDCl_3_, indicating that these molecules undergo decreased motion due to aggregation in this solvent. The stacking propensities of the macrocycles were assessed by comparing absorption spectra measured in different solvents: in CCl_4_ the five compounds adopt the same helical tubular assembly but show different stacking strength, and the aggregation of the macrocycles is interrupted in high polarity solvents. The helical stacking of the macrocycles is also confirmed by CD spectra in CCl_4_, obtained at different temperatures, where induced CD activity could be seen in the UV-Vis bands of the chromophoric backbone, with maxima between 295 nm and 350 nm and minima around 275 nm (Figure 17), indicating an effective translation of the chirality from the amino acidic side chains into the supramolecular structure as a whole. The temperature-dependent nature of the CD traces is fully in line with a supramolecular system assembled under thermodynamic control.

## 3. Conclusions

We have reviewed most recent, prominent examples of organic NTs, in which chirality is expressed in various forms. Many different molecular shapes and forms have been used for the construction of the described structures. We have focused on nanostructures assembled using weak noncovalent interactions; it is note worthy, however, to see that some of the proposed structures are stable in polar solvents, such as in water in physiological conditions. It is somehow surprising to notice that, on most occasions, chiral properties have not been fully developed and translated into new materials properties. We think, therefore, it is safe to say to the many researchers involved that the future is bright in this field of research, and that exciting new properties will be reported in the near future.
